# Laparoscopic Liver Resection by Distance Mentoring – Trinidad to Barbados: A Report

**DOI:** 10.7759/cureus.5796

**Published:** 2019-09-28

**Authors:** Sahle P Griffith, Shamir O Cawich, Marlon Mencia, Vijay Naraynsingh, Neil W Pearce

**Affiliations:** 1 Surgery, Queen Elizabeth Hospital, Bridgetown, BRB; 2 Surgery, University of the West Indies, St. Augustine, TTO; 3 Surgery, Medical Associates Hospital, St. Joseph, TTO; 4 Surgery, Southampton University Hospital National Health Service (NHS) Trust, Southampton, GBR

**Keywords:** liver, resection, minimally invasive, laparoscopic, hepatic, caribbean, trinidad, distance mentoring

## Abstract

Laparoscopic liver resections require advanced laparoscopic skill sets. In the Caribbean, a unique situation exists where centers of excellence for liver resections exist, but surgeons who are trained in advanced laparoscopic surgery are not available throughout the region. Therefore, many patients who are candidates for liver resection in the Caribbean do not have the opportunity to receive laparoscopic operations.

We report a case of distance mentoring using readily available, inexpensive equipment to complete a laparoscopic liver resection, mentored by an expert hepatobiliary surgeon. It may be considered, in special cases, as a way to increase the availability of laparoscopic operations. We acknowledge that there are many limitations to the use of this technology and we discuss the pros and cons of distance mentoring for this purpose.

## Introduction

The laparoscopic approach to liver resections has become widely accepted as the standard of care. However, these operations are technically demanding and require advanced laparoscopic skill sets. A unique situation exists in the Anglophone Caribbean where regional centers of excellence for liver resections were established in 2010 [1], but there are few surgeons with advanced laparoscopic training across the region [2]. In addition, Caribbean Island nations are separated by miles of ocean that limit the movement of patients, surgeons, and equipment. Therefore, many patients in the Caribbean who are candidates for liver resection do not have the opportunity to access laparoscopic liver resections. 

We report a case of laparoscopic liver resection through distance mentoring using readily available, inexpensive equipment. In this situation, one subspecialty trained surgeon encouraged and facilitated another with less experience in a particular operation to be guided through and complete advanced procedures in their own health care setting, without being physically present in the operating room. In special cases, it may be considered as a way to increase the availability of laparoscopic operations. We acknowledge that there are many limitations to this use of technology and we discuss the pros and cons of distance mentoring for this purpose.

## Technical report

**Figure 1:**
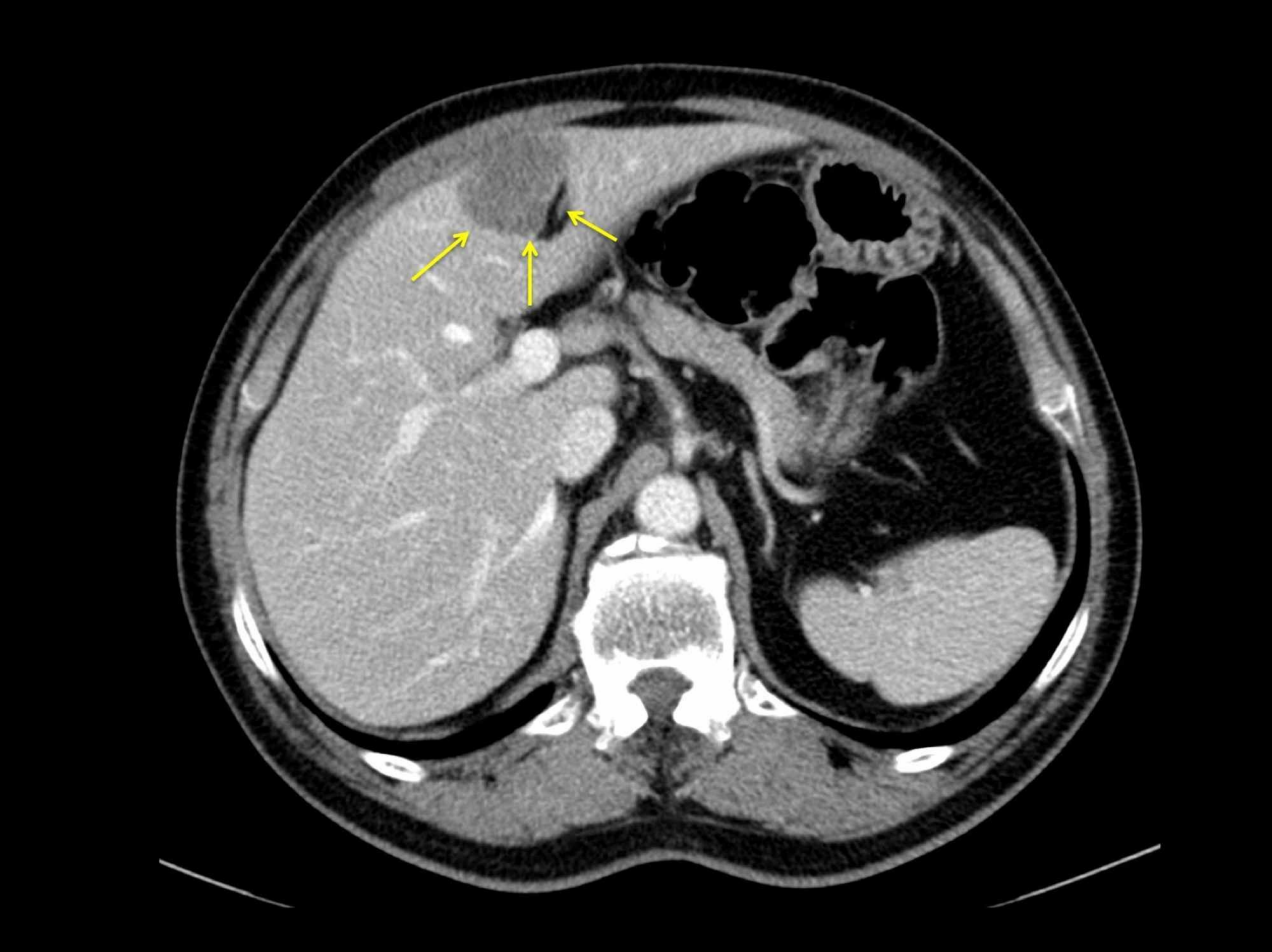
A staging computed tomography scan revealed a 4 cm metastatic deposit in the left lateral segment of the liver

A 65-year-old woman presented to a surgeon in Barbados complaining of constipation. A colonoscopy showed an exophytic lesion in the sigmoid colon, confirmed to be a moderately differentiated adenocarcinoma on biopsy. A staging computed tomography (CT) scan revealed a 4 cm metastatic deposit in segments II/III of the liver (Figure 1). The remnant liver was free of disease and no other metastases were present.

**Figure 2:**
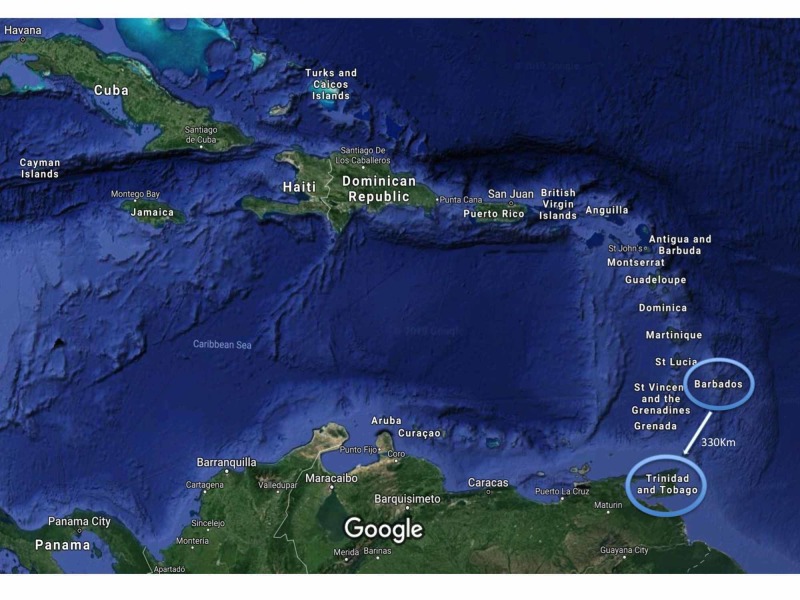
A map of the Caribbean region using Google Satellite Maps® (http://www.google.com/earth/) demonstrating the large footprint with many small island nations separated by the Caribbean Sea The referring surgeon was located on the island of Barbados and the distance mentor was located at the nearest hepatobiliary referral centre in Trinidad and Tobago, 330 km away.

The patient’s condition was discussed at a multidisciplinary team meeting and the decision was made to offer her a laparoscopic sigmoid colectomy with synchronous left lateral sectionectomy. However, there was no hepatobiliary surgeon in Barbados. Therefore, the patient was referred to the nearest hepatobiliary centre, 330 km away in Trinidad and Tobago (Figure 2). The patient, however, was reluctant to travel overseas for surgery, and the hepatobiliary surgical team was not able to travel to Barbados. We faced a treatment dilemma. 

The referring Barbadian surgeon was an accomplished, fellowship-trained expert laparoscopic surgeon with significant colorectal practice but with limited experience in laparoscopic liver resections. After much discussion between the surgeons, video consultation with the patient, and approval by the administrative ethical board of both facilities involved, a decision was made to proceed to surgery, with the Trinidad hepatobiliary surgeon mentoring the colorectal surgeon in performing a laparoscopic left lateral sectionectomy without being physically present in the operating room.

The hepatobiliary surgeon organized a protected time in order to be virtually present during the entire operation. He was seated in an office in Trinidad 330 km away with two computers equipped to communicate in real-time using FaceTime® video chat software (Apple, Inc., Cupertino, CA, USA). 

**Figure 3:**
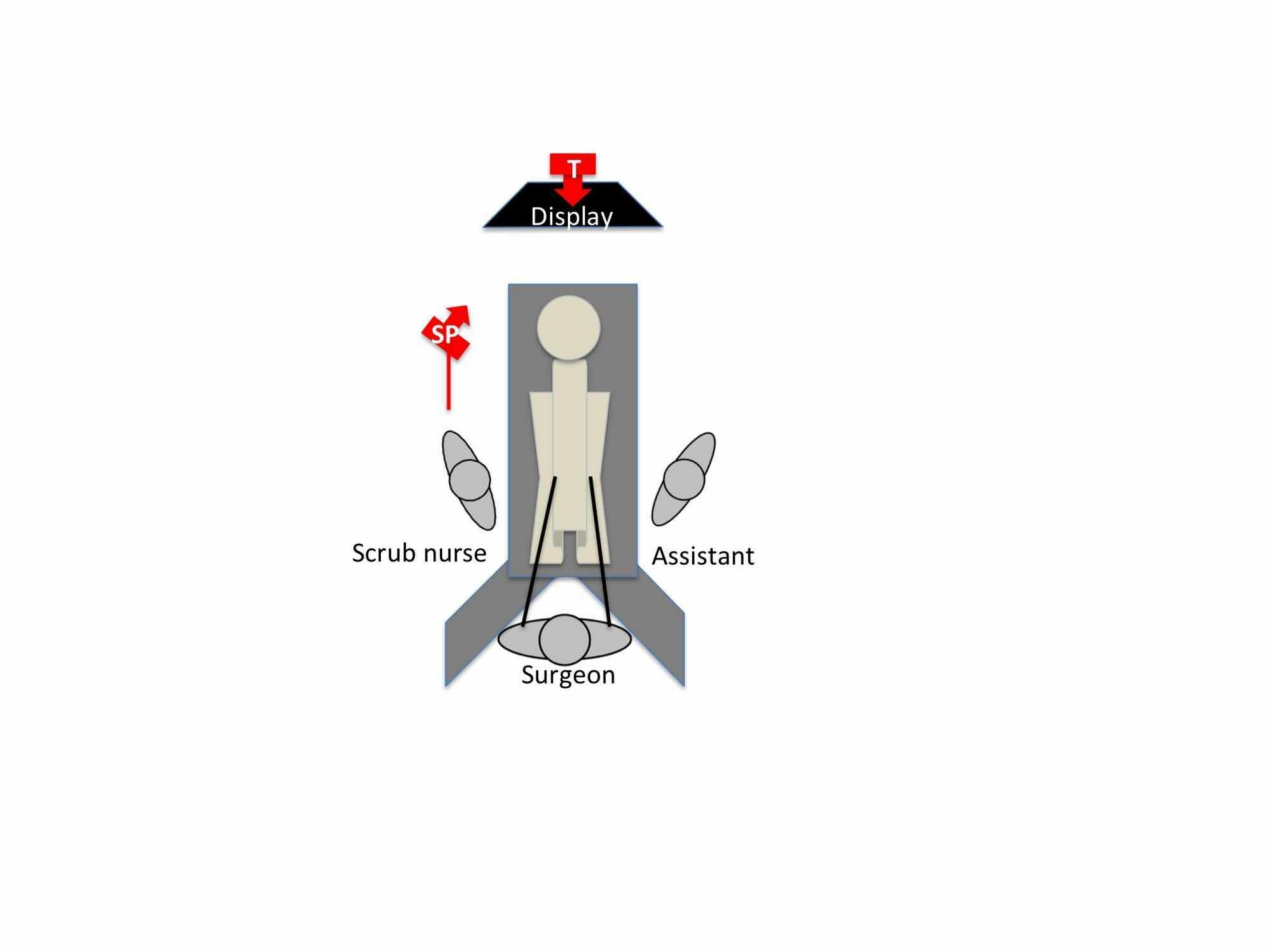
Operating room setup for distance mentoring The primary monitor was placed above the patient’s head and the surgeon stood between the patient’s legs with the patient in Lloyd-Allen Davies position. One Apple iPhone® was placed on the laparoscopic stack facing backward (red arrow - T) for the mentor to view the external operating field, including the operating surgeon’s port placement and instrument handling. A second iPhone (red arrow - SP) was fixated onto an intravenous (IV) stand and positioned over the surgeon's left shoulder facing the primary monitor. SP: smart phone; T: tablet

In Barbados, a thorough informed consent process was undertaken with the patient. The patient was prepared for anaesthesia and taken to the operating room. The operating room was organized in the usual manner for the primary surgeon to comfortably visualize his working monitors. For the liver resection, the primary monitor was placed above the patient’s head and the surgeon stood between the patient’s legs with the patient in the Lloyd-Allen Davies position (Figure 3).

**Figure 4:**
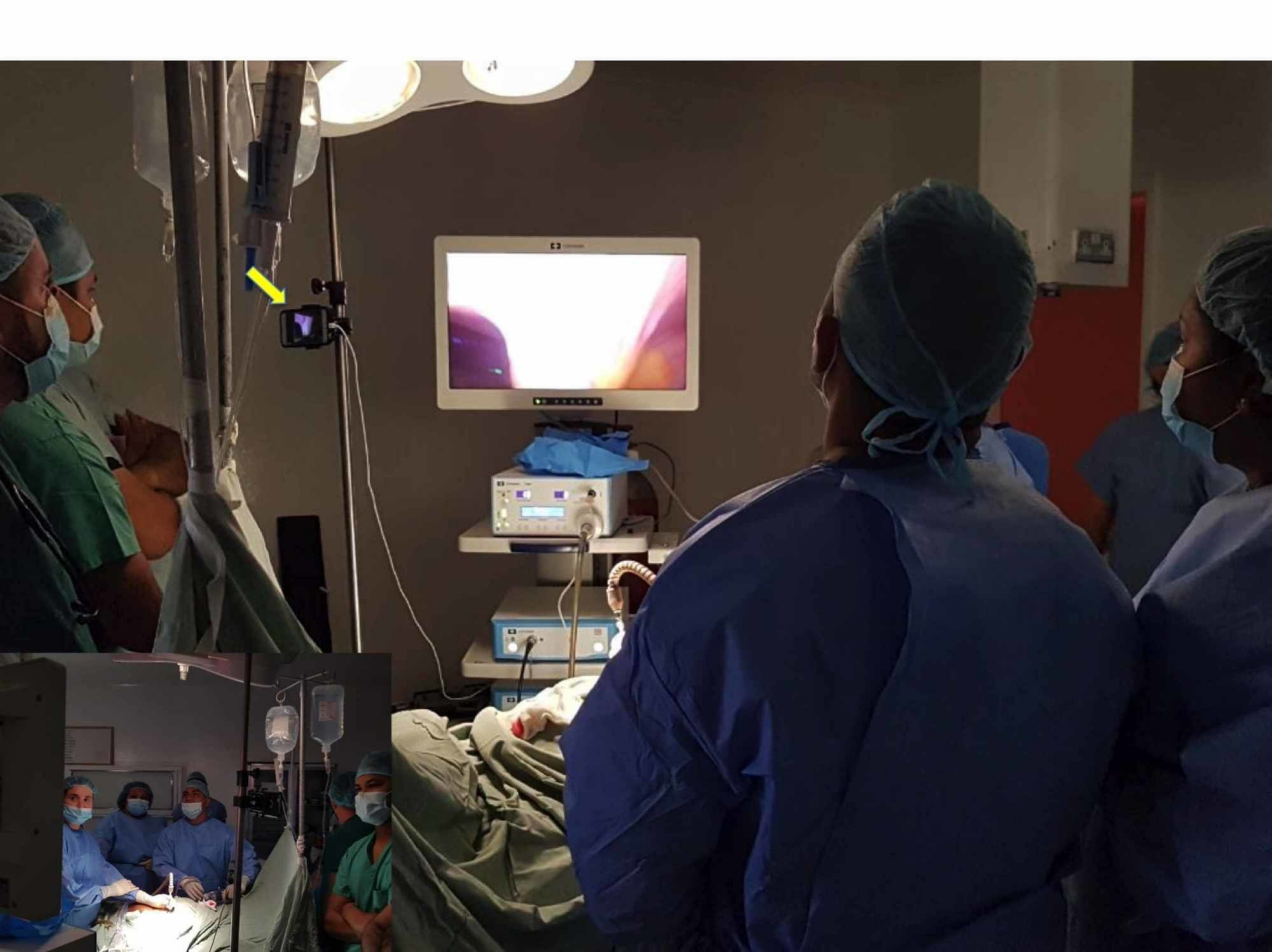
Setup for distance mentoring An Apple iPhone®^ ^was fixated onto an intravenous (IV) stand (yellow arrow) and positioned over the surgeon's left shoulder in order not to obstruct the surgeon’s view. This iPhone faces the primary monitor and is used to stream the live feed to the remote mentor, providing him with a real-time view of the laparoscopic feed.

Proprietary video conferencing equipment was not available in this resource-poor environment so we used two personal Apple iPhones® (Apple, Inc., Cupertino, CA, USA) equipped with FaceTime to stream a live feed to the remote mentor. The first iPhone was fixated onto an intravenous (IV) stand and positioned over the surgeon's left shoulder facing the primary monitor (Figure 4). It was specifically placed in this location in order not to obstruct the surgeon’s view.

**Figure 5:**
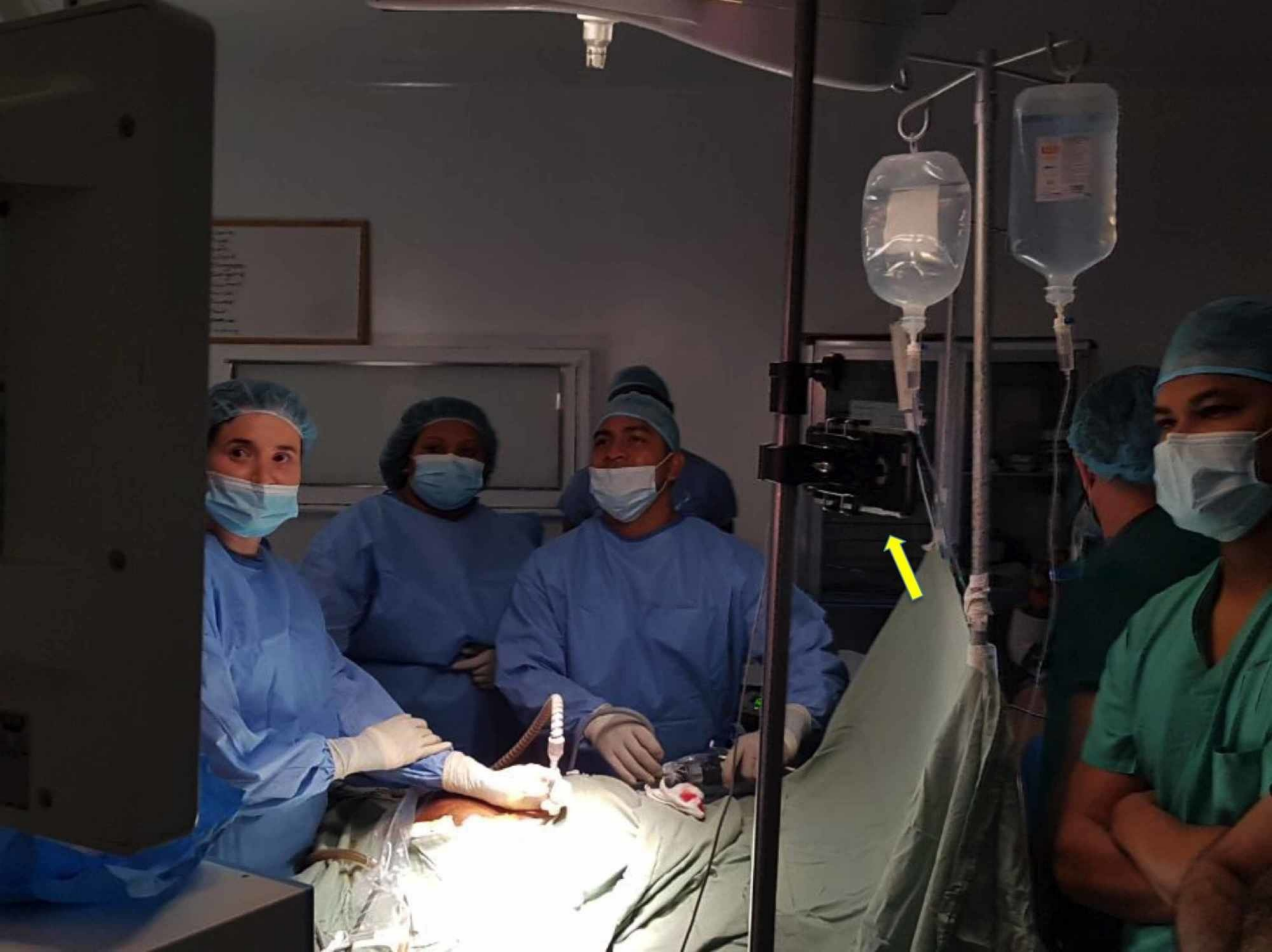
Setup for distance mentoring A second iPhone^® ^was placed on the laparoscopic tower and faced backward to view the external operating field. This smartphone provided the mentor with a real-time view of the operating field, the surgeon’s port placement, and instrument handling.

Another smartphone was placed on the laparoscopic stack facing backward to view the external operating field (Figure 5). This setup provided the mentor with a real-time view of the laparoscopic feed, plus the operating surgeon’s port placement and instrument handling. 

Communication was established and the operation proceeded. The colorectal surgeon completed the laparoscopic sigmoid colectomy uneventfully. Attention was then turned to the liver. With instruction from the hepatobiliary surgical mentor who was observing the live operation by FaceTime, the resection margins were chosen using the umbilical fissure as an anatomic landmark to ensure > 1 cm gross margins. Glisson’s capsule was then scoured and the parenchyma was transected using a combination of ultrasonic dissectors and staples. The left lateral sectionectomy was completed uneventfully. 

The patient was returned to the high-dependency unit for monitoring. Thereafter, the recovery period was uneventful, and she was discharged from the hospital on Day 4 post-surgery. Histologic assessment confirmed the presence of a metastatic deposit in the left lateral section with clear resection margins. 

## Discussion

Laparoscopic surgery in the Anglophone Caribbean is still relatively novel, having only gained popularity at the turn of the 21st century after the repatriation of fellowship-trained surgeons with experience in advanced laparoscopy [2-3]. However, while advanced laparoscopy is available in select territories, it is still not universally available across the region. 

This is because most Caribbean countries are small island states that are geographically separated by the Caribbean Sea. Most of the islands are independent, with their own governments, health care systems, regulatory medical councils, and a small cadre of surgeons with experience in laparoscopy. Many of these countries are not able to attract subspecialists to their workforce due to the low case volumes and lack of support services. In most cases, it is not feasible for subspecialists to visit these areas because their services are limited by equipment deficiencies, insufficient support services, and regulations imposed by each country’s medical council. Inter-island patient transfers are also difficult due to unreliable air/sea transportation and the absence of financial agreements to facilitating patient transfers. Also, a transferred patient is often removed from social and family support. This produces a situation where patients who are in need of subspecialty services are not able to access these services in a timely fashion. This is evident in patients with colorectal liver metastases; most patients with colorectal liver metastases who are candidates for resection are unable to access these services and so their oncologic treatment is limited to best medical management only [4-5]. 

This situation extends to laparoscopic liver resections as well. In this environment, laparoscopic liver resections are only performed in Trinidad and Tobago, Jamaica, and the Bahamas [1, 3, 5-6], where there are active hepatobiliary teams (Figure 1). Some of the other Caribbean countries have competent surgeons who may not have formal training in liver resections but possess the necessary advanced laparoscopic skill sets to complete laparoscopic liver resections. In these cases, these surgeons may be taught to perform liver resections through surgical mentoring. The concept of mentoring in surgery is well-accepted in surgical training [7]. This is where an experienced surgeon coaches a less experienced mentee who is in an earlier phase of their career. In this report, we described a case in which a laparoscopic surgeon was mentored in liver resections. Although this is not unusual, achieving this by distance mentoring was novel. In this case, logistic issues prevented the patient from traveling to the nearest hepatobiliary centre and the hepatobiliary surgical team from visiting Barbados to perform the laparoscopic liver resection in a timely fashion. Our solution was distance mentoring. 

We acknowledge that there are limitations to this concept that include acquiring a working knowledge of the technology, lack of direct interpersonal contact, inability to demonstrate technical tricks/tips, inability to intervene at operation, mentor availability, mentor-mentee matching, and case selection. We discuss the methods used to overcome these barriers in the hope that distance mentoring may be considered in other developing countries with similar challenges.

Working knowledge of technology

The use of two-way communication using internet protocols for videotelephony in medicine is not new. It has been used before for virtual consultations, diagnostics, medical education, and transmission of medical images [8-10]. The use of videotelephony for distance mentoring is relatively new, but it is limited by cost, equipment complexity, signal latency, and data transfer speeds.

Proprietary video conferencing equipment to facilitate real-time communication does exist, but it is expensive and was unavailable in our resource-poor setting. Therefore, we made use of free software wherever possible, such as OsiriX® DICOM imaging software (Pixmeo, Geneva, Switzerland) to view scans, Dropbox® (Dropbox, Inc., San Francisco, CA, USA) to share clinical data, and FaceTime to communicate in real-time during operations. These are available free on the internet and so are affordable, even in the resource-poor Caribbean setting. Dropbox® is a proprietary file-sharing software that is freely available on the internet. FaceTime is proprietary software in Macintosh® models (Apple, Inc., Cupertino, CA, USA) after 2011 that establishes a high-definition video connection between two supported devices. Most persons already have the software installed on their smartphones for personal use and so this is not an additional expense.

Communication hardware and software add a level of complexity that many users may not fully understand. Therefore, surgeons may be reluctant to use the technology because of a preconceived notion that it is unreliable, ineffective, or inoperable. To overcome this, it may be useful to provide targeted training to the surgeons or to have information technology (IT) support teams available during the operation.

In order to facilitate videotelephony, smartphones run codec software to compress audio-video streams and organize them into labeled digitalized packets [11]. These labeled digitalized packets are transmitted using internet protocols to any location with an internet connection. However, time is required for data compression and transmission. Depending on the data size, internet service bandwidth, and connection quality, there is a delay between transmitting and receiving data that is known as signal latency [10]. Although most modern video-enabled smartphones use MPEG-4 codecs that transmit high-definition audio-video signals at 2 megabytes per second, signal latency still becomes distracting when the signal latency exceeds 200 milliseconds [10]. Therefore, it is important to establish a reliable network connection beforehand because connectivity problems intraoperatively will exacerbate surgeon and mentor fatigue and frustration, especially in these high-stress situations. 

Lack of direct interpersonal contact

Face-to-face interactions help to develop interpersonal relationships. A shortcoming of the distance mentoring model is the absence of direct interpersonal contact. 

In our model, the participants had already established a working relationship by operating together in traditional settings before engaging in distance mentoring. They also had occasional face-to-face meetings at regional conferences and educational symposia. Therefore, both participants had established a trust-based relationship and were aware of each other’s skillsets, capabilities, and judgment. We recommend that all persons who wish to engage in distance mentoring should have a working relationship already established so that there are mutually clear expectations from the exercise.

Of course, there are still inherent limitations. The human brain exploits parallax from differing views in each eye to carry out stereopsis, allowing depth perception and distance estimation [12]. When a smartphone with a single feed camera is used as a communication device, these faculties are not available. The end-user should be aware of this limitation since our model using a smartphone was placed at an angle facing the visual feedback monitor, interrupting parallax. It could be argued that laparoscopic surgeons have already developed the ability to compensate for this by virtue of the two-dimensional view on laparoscopic monitors. However, this also gives added value to our setup where a second smartphone was used to view the operating field. 

Face-to-face interactions also allow us to have eye contact and to exploit non-verbal communication or “body language” to perceive intent, attitudes, and attention. We should be aware of the potential for distance mentoring to transmit confusing visual cues to the users due to the loss of direct eye contact and inability to use “body language.” For example, there may be the impression that a user may be avoiding eye contact or using inappropriate body language cues that may impair remote communication. 

Users should be aware of appearance consciousness - the psychological need to present an acceptable on-screen appearance when one knows they are on camera [13]. It may enhance performance for some individuals who raise their game when they know they are being watched, but it can also be counterproductive by distracting the operating surgeon, preventing concentration on the operative tasks. Laparoscopic surgeons may already be used to appearance consciousness since the operating room staff are constantly viewing all steps of laparoscopic operations. 

Inability to intervene intraoperatively

Videotelephony is well-suited for data transfer, medical education, and virtual consultations [8-10], but it is not ideal for teaching technical skills. Since the opportunity to “take over” the operating instruments does not exist, the distance mentor cannot demonstrate technical maneuvers, tips, and tricks during the operation. Therefore, the mentors should be experienced teachers who are able to verbalize instructions in a clear and effective manner. 

This is a major limitation in the event of an intraoperative complication. This should be a point of detailed discussion between the surgeon, the mentor, and the patient before proceeding with distance mentoring. We believe that this discussion should be a part of the mandatory preoperative video consultation and all participants should be comfortable with this limitation. 

We should always consider patient safety first, especially since the mentor would not be able to intervene in the surgery. We believe that it is mandatory that the operating surgeon should be able to perform the operation via the open approach and convert to open surgery, if necessary, to address any complications that may arise. It may also be beneficial to have predetermined “red lines”, such as a threshold operating time or blood loss, at which point the case should be converted if crossed. 

Mentor-mentee matching

For this exercise to be successful, the mentor and the operating surgeon should have a good working relationship. To achieve this, we recommend that there should be a period of traditional proctorship with the mentor physically present in the operating room before distance mentoring is pursued. This would allow the mentor to determine whether the mentee would be able to effectively manage a potential complication without the mentor being physically present in the operating room. 

The mentors’ personalities would also contribute to the success of this exercise. The ideal surgical mentor should be one who is experienced, well-trained, and willing to help junior colleagues to develop professionally [7, 14-15]. The mentor should be easily accessible and able to dedicate time to addressing the mentee’s needs [7, 14-15]. One should also take into account other factors that may influence the relationships, such as generational differences [7], cultural factors [16], gender [16-17], and work ethic [7, 16]. Although these qualities are important for successful mentorship in general, specific to distance mentoring, it is important for the mentor to have patience, to be emotionally intelligent, to have good verbal communication skills, and to be fluent in the same language as the mentee. 

It is equally important for the operating surgeon to be experienced in handling laparoscopic instruments and performing other laparoscopic operations. In our case, the operating surgeon regularly performed laparoscopic colectomies but had no experience in laparoscopic liver resections. Therefore, the surgeon would have already been used to the hand-eye coordination and laparoscopic skill sets, only now applying them to a new procedure. 

Mentor availability

In our model, the mentor blocked off time to provide a virtual presence to ensure distance mentoring for the entire duration of the operation. In this model, the mentor performed this service at no cost, but this may not be sustainable because potential mentors will always have competing demands that limit their availability. Therefore, thought should be given to developing reward systems to encourage mentoring [14, 18]. This may include explicit funding, providing protected time for mentoring activities, or incentives, such as academic promotions and/or earning leave time [7, 14-15, 18]. Whatever is chosen to encourage mentors should be clearly discussed beforehand so that expectations are clear. 

Case selection

Mature surgical judgment is required to select appropriate cases for distance mentoring. Complex cases are not well-suited for distance mentoring. We recommend selecting straightforward cases for this exercise. In this report, we chose a left lateral sectionectomy - but a trisectionectomy would have been inappropriate due to procedural complexity, high technical demands, need for more complex instrumentation, and a greater potential for complications.

There should be a well-thought-out planning period during which the mentor and mentee discuss cases in detail prior to operation. This should include a detailed review of clinical information, relevant investigations, and scans. We believe that distance consultations should also be undertaken to familiarize patients with distance mentors. This gives all team members a chance to be familiarized with each other and allows complete disclosure and detailed discussion. This is an invaluable adjunct that should be utilized.

Maintaining safety

A priority that must be observed is non-maleficence. This is particularly important since the mentee surgeon would not have a comparable experience to the mentor and the mentor would not be physically in the room to take over the operation. In this situation, patient safety must be a two-way process. This requires the two surgeons to be very familiar with each other - knowing each other’s abilities and limitations, as well as also trusting each other’s skillsets. The surgeons should have sufficient self-awareness and willingness to adapt to each other’s abilities. They should also be able to understand the limitations of technology, available equipment, and assistance. We do not believe that this technique will work with all surgeons or surgical situations. It requires careful case selection and excellent communication preoperatively and during the procedure. 

The mentor and mentee must build a bond of confidence in each other, which will evolve as cases progress, depending on the success of the communication and working relationship between the two. In some cases, it may become rapidly apparent that this relationship will not function effectively. In cases where there is a tenuous mentor-mentee relationship, the operation should not proceed. In addition, there should be thorough discussion and agreement before the operation begins that in the event of a disagreement, the expert mentor should have the final say to maintain patient safety. 

There also needs to be a thorough case discussion before the operation, describing the steps involved, and considering the equipment that is available and might be required, along with strategies for coping with adverse intraoperative events, such as unexpected findings, significant haemorrhage, or iatrogenic injury to other structures or organs. All of these need to be considered and planned for in order to maintain patient safety.

A specific plan should exist in the event of complications. This would be complication-specific. For example, the commonest intraoperative complication during a left lateral sectionectomy is excessive bleeding. In this case, the preexistent plan would be first to attempt to apply a Pringle’s maneuver and compress the liver using a laparoscopic "closed book" technique. This usually controls bleeding from low flow veins within the liver. However, in the case that this was unsuccessful, conversion to open surgery would be performed and the bleeding controlled with manual compression and use of staples. 

Informed consent

In keeping with the informed consent principles, full disclosure is required if there is an intention to utilize distance mentoring. The patient must be aware of the arrangement, including the potential risks, benefits, and available alternatives. They should also be given the opportunity to meet with the surgical mentor by videotelephony in order to discuss the procedure and have their questions answered by both the distance mentor and the mentee surgeon. 

Medicolegal considerations

Although we have shown that it is feasible to perform laparoscopic liver resections by distance mentoring, we recognize that the technical aspect has developed faster than the medicolegal aspect. Some potential medicolegal issues that must be thought out are physician licensure, malpractice indemnity coverage, data loss/privacy breaches, responsibilities of mentors, and the quality of patient-physician relationships.

Distance mentoring now enables the mentor to participate in care delivery across international borders, potentially bypassing registration procedures in other jurisdictions. In some countries, there are accelerated registration pathways to facilitate telemedicine [19-20], but the legislation to govern technology does not exist in the Caribbean states.

It is clear that the primary surgeon who performs the operations has legal and ethical responsibilities to the patient. However, there is ambiguity when considering the legal and ethical responsibilities of the surgical mentor who interacts with the mentee surgeon while the patient is anesthetized and has “hands-off” interactions during the operation. This ambiguity also filters down to malpractice indemnity coverage, and in the Caribbean, the legislation does not exist for these issues. 

There is also the ever-present risk of data capture/privacy breaches during distance mentoring. This is an important consideration because the healthcare provider has the ultimate responsibility to safeguard patients from emotional, spiritual, social, and material harm [19]. This may be achieved by using secure data transmission networks, firewall protection, and encryption protocols [19]. It is also important to disclose to patients that privacy and confidentiality cannot be guaranteed during operative videotelephony, challenging traditional concepts of privacy and confidentiality.

## Conclusions

Distance mentoring, if carefully and effectively administered, may be an option to increase access to advanced laparoscopic procedures across the region. This requires the institutional and medical culture to evolve into one that actively supports mentoring.

More research is needed to explore medicolegal implications, define desirable mentee traits and expectations, overcoming generational and cultural differences, and methods to overcome current barriers to effective mentorship (such as time constraints and lack of qualified mentors). 
